# Biologically Active Sheep Colostrum for Topical Treatment and Skin Care

**DOI:** 10.3390/ijms25158091

**Published:** 2024-07-25

**Authors:** Kinga Kazimierska, Ilona Szabłowska-Gadomska, Stefan Rudziński, Katarzyna Kośla, Elżbieta Płuciennik, Łukasz Bobak, Aleksandra Zambrowicz, Urszula Kalinowska-Lis

**Affiliations:** 1 Department of Cosmetic Raw Materials Chemistry, Faculty of Pharmacy, Medical University of Lodz, 90-419 Lodz, Poland; kinga.kazimierska@student.umed.lodz.pl; 2 Laboratory for Cell Research and Application, Center for Preclinical Research and Technology, Medical University of Warsaw, Banacha 1b, 02-097 Warsaw, Poland; ilona.szablowska-gadomska@wum.edu.pl (I.S.-G.); stefan.rudzinski@wum.edu.pl (S.R.); 3 Department of Molecular Carcinogenesis, Medical University of Lodz, 90-419 Lodz, Poland; katarzyna.kosla@umed.lodz.pl; 4 Department of Functional Genomics, Medical University of Lodz, 90-419 Lodz, Poland; elzbieta.pluciennik@umed.lodz.pl; 5 Department of Functional Food Products Development, Wroclaw University of Environmental and Life Science, 51-640 Wrocław, Poland; lukasz.bobak@upwr.edu.pl (Ł.B.); aleksandra.zambrowicz@upwr.edu.pl (A.Z.)

**Keywords:** antioxidant, cosmetic ingredients, fibroblasts, keratinocytes, gene expression, proliferation, scar test, sheep colostrum, skin diseases

## Abstract

Colostrum is gaining popularity in cosmetic products. The present study compared the composition and selected biological properties of colostrum from Polish sheep (colostrum 1) and Swiss sheep (colostrum 2), particularly those that can affect healthy or diseased skin. The antioxidant activity of the colostrums was measured using ABTS and DPPH assays. The effect on the proliferation of human skin fibroblasts, neonatal epidermal keratinocytes, and human diabetic fibroblast (dHF) cells isolated from diabetic foot ulcers was also assayed in vitro by MTT and Presto Blue tests, respectively. The colostrum simulated dHF cell proliferation by up to 115.4%. The highest used concentration of colostrum 1 stimulated normal fibroblast proliferation by 191.2% (24 h) and 222.2% (48 h). Both colostrums inhibited epidermal keratinocyte viability. The influence of the colostrums on the expression of genes related to proliferation (*Ki67*) and immune response (*IL-6*, *PTGS-2*, *TSG-6*) in dHF cells were compared. Colostrum 1 increased the rate of wound closure (scar test). Analysis of total fat, protein and fatty acid content found the Polish colostrum to be a richer source of fat than the Swiss colostrum, which contained a larger amount of protein. Both colostrums exhibit properties that suggest they could be effective components in cosmetic or medicinal formulations for skin care, especially supporting its regeneration, rejuvenation, and wound healing.

## 1. Introduction

Colostrum, the so-called first milk, is a thick, yellow secretion of the mammary glands produced by mammals in the first few hours after giving birth. Sheep colostrum and mature milk provide essential nutrients and bioactive components critical for the growth and development of newborn lambs prior to weaning [1]. The composition and biological activities of milk vary widely among species and breeds, being influenced by genetics, environmental conditions, and dietary factors [2]. However, the key functional elements in milk are proteins, which can be categorized into three primary groups: caseins, whey proteins, and milk fat globule membrane proteins [2,3,4]. Among these, the predominant proteins in milk are caseins, which provide crucial amino acids necessary for the growth of newborns [4,5]. Milk fat globule membrane proteins enable the transport of fats and proteins, cell signaling, metabolic regulation, and other key biological functions [6,7]. Whey proteins, which constitute about 20% of total milk protein content [8], are essential for providing neonatal protection via antioxidant properties, immune-enhancing effects, and anti-inflammatory activities [3,9].

Colostrum is also notably rich in fats, carbohydrates, and proteins, and its complex composition provides essential micronutrients (vitamins and minerals), antimicrobial agents (including lactoferrin and lysozymes), and growth factors [2,10]. It contains essential minerals including selenium (Se), which are vital for preventing nutritional shortfalls in nursing lambs; without these, they could suffer myodegenerative conditions. For example, selenium deficiency can result in white muscle disease [2,11,12,13]. Furthermore, other important minerals in colostrum, such as magnesium (Mg), manganese (Mn), zinc (Zn), and copper (Cu), have significant roles in protecting the integrity of cellular membranes from oxidative stress [2,13,14].

However, there is little research on sheep colostrum compared to bovine colostrum [15,16,17,18,19,20]. The present study compares the content and certain biological activities between colostrum of Polish and Swiss origin. It is hypothesized that differences in the environment between Poland’s continental climate and Switzerland’s alpine regions may influence the nutritional and biological profiles of colostrum, and thus its potential therapeutic value. The analysis focuses on the total protein level and lipid compositions, which are vital for its therapeutic efficacy, particularly in skin care and human health.

The study examines the impact of colostrum on the growth of diabetic human fibroblast cells, a critical area of research given the challenges associated with wound healing in diabetic conditions. This aim is to explore how colostrum can potentially accelerate cell regeneration and improve wound healing outcomes in diabetic patients.

The research explores the capacity of colostrum to promote the growth of fibroblasts and keratinocytes, key cells involved in skin health and wound healing, and assesses its antioxidant properties. It also examines its potential to enhance tissue repair and manage scar formation, crucial aspects of post-operative care and injury recovery, in scar test models.

The proposed in vitro tests aim to check whether colostrum will be a good choice as an active component in cosmetic and medicinal preparations with rejuvenating properties, delaying skin aging, regenerating and accelerating wound healing, etc. Moreover, parallel tests on colostrum from two sources (Polish and Swiss) will allow us to compare their properties and indicate the better one.

## 2. Results

### 2.1. Fat, Protein and Fatty Acid Analysis

Noticeable differences in total fat and protein content were found between freeze-dried colostrum 1 and 2 (Table 1). Colostrum 2 had higher protein content (43.68%) than colostrum 1 (34.14%), while colostrum 1 had a much higher fat content (38.05%) than colostrum 2 (26.63%).

Distinct differences in the concentrations of specific fatty acids were noted between colostrum 1 and 2 (Table 2). Colostrum 2 was characterized by a significantly higher PUFA (polyunsaturated fatty acids) content and a slightly higher SPA (saturated fatty acids) content than colostrum 1, but a significantly lower MUFA (monounsaturated fatty acids) content than colostrum 1.

The predominant saturated fatty acids (SFA) in both colostrums were palmitic, myristic, and stearic acids. Both colostrum 1 and colostrum 2 contained almost identical amounts of palmitic acid, 37.41% and 37.14%, respectively, while colostrum 2 had slightly higher levels of myristic acid (11.17% vs 13.04%) and stearic acid (10.65% vs 11.86%). Caprylic and capric acids were absent from colostrum 1, but were present in very small amounts in colostrum 2: 0.36% and 1.35%, respectively.

Among monounsaturated fatty acids (MUFA), both oleic acid and palmitoleic acid were more abundant in colostrum 1 than in colostrum 2. The oleic acid content was 29.11% and 23.46% in colostrum 1 and 2, respectively, and the palmitoleic acid content was 2.02% and 1.17%.

Polyunsaturated fatty acids (PUFA), including linoleic acid (LA), conjugated linoleic acid (CLA), and alpha-linolenic acid (ALA), accounted for only 4.86% and 6.62% of all fatty acids contained in colostrum 1 and 2, respectively. Among these, LA predominated, amounting to 2.98% and 3.97% in colostrum 1 and 2, respectively (Table 2).

### 2.2. ABTS and DPPH Free Radical Scavenging Activity Assays

In the DPPH assay, colostrum 1 and colostrum 2 exhibited moderate antioxidant activity, with EC_50_ values of 0.988 mg/mL and 0.806 mg/mL, respectively (Table 3). In the ABTS assay, both colostrum 1 and 2 demonstrated around two times stronger anti-radical activity than anti-DPPH activity, with EC_50_ values of 0.515 mg/mL (after 30 min) and 0.477 mg/mL (after 40 min) for colostrum 1, and 0.455 mg/mL (after 30 min) and 0.424 mg/mL (after 40 min) for colostrum 2. The antioxidant potential was around one hundred times lower than for the antioxidative standard used, i.e., ascorbic acid, for which the EC_50_ value was 0.0053 mg/mL. Colostrum 2 exhibited slightly higher activity than colostrum 1, determined by both DPPH and ABTS assay.

**Figure 1:**
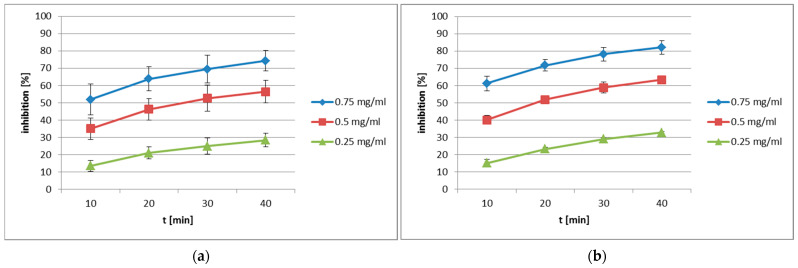
Percentage of ABTS radical inhibition by colostrum 1 (**a**) and colostrum 2 (**b**) over time (n = 3).

Additionally, the percentage inhibition of the ABTS radical by colostrum 1 and 2 (0.25 mg/mL, 0.5 mg/mL, and 0.75 mg/mL) was measured every 10 min over a period of 40 min (Figure 1). The greatest increase in inhibition was observed in the first 10 min: colostrum **1** demonstrated 13.5%, 35.0%, and 51.8% inhibition at concentrations of 0.25 mg/mL, 0.5 mg/mL, and 0.75 mg/mL, respectively, while **2** demonstrated 15.1%, 40.1%, and 61.2% inhibition. This increase leveled out between 10 and 40 min for both colostrum **1** and **2**, amounting to only around 20 p.p. for 0.75 mg/mL and 0.5 mg/mL, and around 15 p.p. for 0.25 mg/mL.

### 2.3. Inhibition of Tyrosinase Activity

To investigate the potential skin-whitening effect of colostrum 1 and 2, their ability to inhibit tyrosinase activity was tested using ascorbic acid as a reference standard. Samples with a concentration of 1 mg/mL were assessed (Table 4). Colostrum 1 demonstrated weak inhibition (23.4%), around four times lower than ascorbic acid; however, this value was approximately three times higher than for colostrum 2 (7.0%).

Although this study found that colostrum 1 has some inhibitory activity, the level was not significant compared to ascorbic acid. Therefore, neither colostrum 1 nor colostrum 2 appear to be a good source of whitening substances for possible skin therapy or care.

### 2.4. Proliferation of Fibroblasts and Keratinocytes

The effect of colostrum 1 and 2 on human skin fibroblast 1BR.3.N and human neonatal epidermal keratinocytes in relation to dose and time was determined by MTT assay.

**Figure 2:**
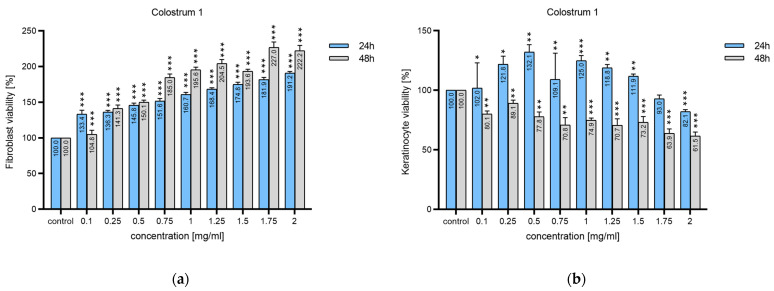
The effect of colostrum 1 on the cell viability of: (**a**) 1BR.3.N human skin fibroblasts; (**b**) human neonatal epidermal keratinocytes (n = 6); *** *p* < 0.001; ** *p* < 0.01, * *p* < 0.05.

After 24-h and 48-h incubation, all concentrations of colostrum 1 significantly increased fibroblast growth in a dose-dependent manner when compared to untreated cells (Figure 2a). The highest increase in proliferation, 91.2% (24 h) and 122.2% (48 h), was observed for colostrum 1 at a concentration of 2 mg/mL.

Human epidermal keratinocytes exhibited varying sensitivity to colostrum 1 (Figure 2b). Incubation for 24 h at low concentrations (up to 0.5 mg/mL) slightly increased cell viability by 32.1%, whereas high concentrations (2 mg/mL) reduced viability by up to 82.1%. After 48 h, the effect of decreasing keratinocyte viability was already evident at the lowest dose of 0.1 mg/mL, and increasing the concentration resulted in additional suppression of keratinocyte cell viability, i.e., a reduction of around 40% at 2 mg/mL.

**Figure 3:**
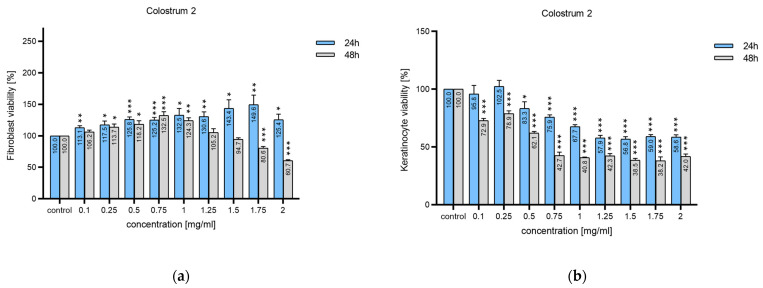
The effect of colostrum 2 on the cell viability of (**a**) 1BR.3.N human skin fibroblasts; (**b**) human neonatal epidermal keratinocytes (n = 6); *** *p* < 0.001; ** *p* < 0.01, * *p* < 0.05.

For colostrum 2, after 24 h of incubation, fibroblast proliferation increased with increasing concentration (Figure 3a). However, a smaller increase (from 13.1% to 49.6%) was measured compared to colostrum 1. Incubation for 48 h resulted in a slight increase in proliferation (around 20%) from 0.25 mg/mL to 1 mg/mL, while incubations with 1.75 mg/mL and 2 mg/mL reduced cell viability by 19.4% and 39.3%, respectively.

Incubation for 24 h inhibited human keratinocyte cell proliferation by 16.7% to 41.4% relative to untreated cells, at concentrations from 0.5 to 2 mg/mL; also, 48-h incubation reduced proliferation by 27.1% to 58.0% compared to controls at concentrations from 0.1 to 2 mg/mL (Figure 3b).

### 2.5. Proliferation of Diabetic Human Fibroblasts

**Figure 4:**
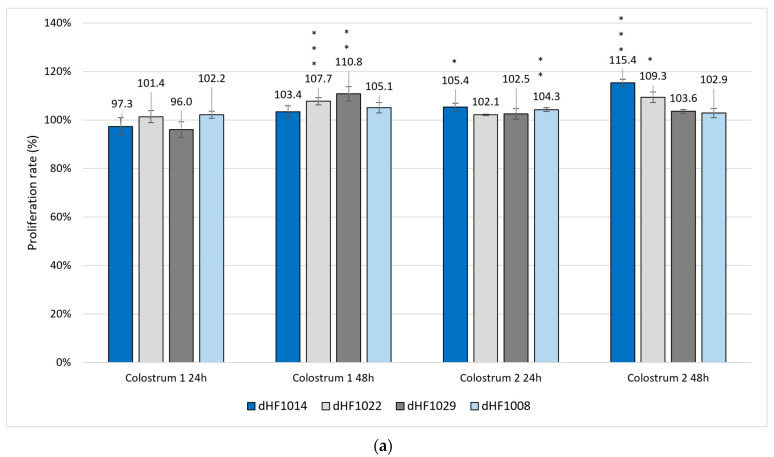

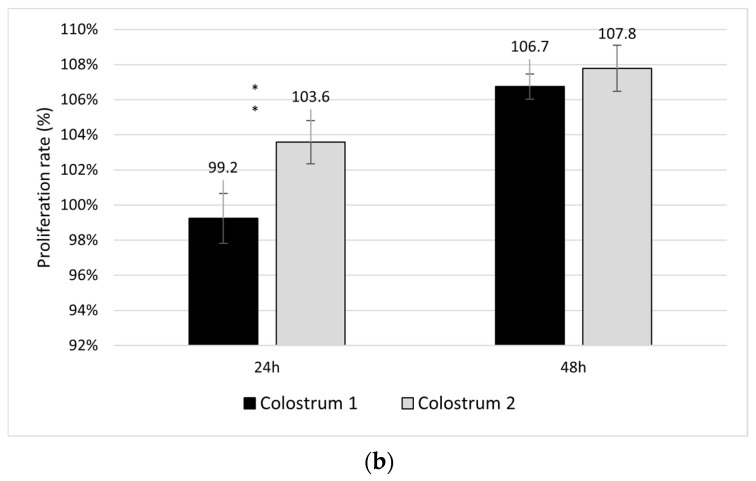
Presto Blue proliferation test results for diabetic human fibroblasts (dHF): (**a**) four different dHF cells samples (n = 3) cultured with either colostrum 1 or colostrum 2; (**b**) cumulative results of Presto Blue proliferation tests (n = 12). Proliferation rate was calculated with a dHF cultured in standard dHF medium as the control. The proliferation rate of stimulated dHF cells ranged from 96.0% (dHF1029—cultured with colostrum 1 after 24 h) to 115.4% (dHF1014 cultured with colostrum 2 after 48 h). SEMs were calculated. The statistical significance was calculated with Fisher’s test and Student’s *t*-test. *** *p* < 0.001; ** *p* < 0.01; * *p* < 0.05.

The effect of colostrum 1 and 2 on diabetic human fibroblasts showed mixed trends depending on the cells sample material; the results are presented in Figure 4. Cells cultured with colostrum 1 after 24 h showed mixed tendencies: the values were either lower than controls, viz. dHF1014 (97.3%) and dHF1029 (96.3%), or slightly higher, viz. dHF1022 (101.4%) and dHF1008 (102.2%). After 48 h in culture with colostrum **1,** all cells showed higher proliferation rates, especially dHF1022 (107.7%, *p* < 0.001) and dHF1029 (110.8%, *p* < 0.01). For colostrum 2, an increase in proliferation rate was observed already after 24 h, with significant results noted for dHF1014 (105.4%, *p* < 0.05) and dHF1008 (104.3%, *p* < 0.01). After 48 h, the cultured cells showed an even higher increase in proliferation rates: dHF1014 (115.4%, *p* < 0.01); dHF1022 (109.4%, *p* < 0.05).

Generally, the dHF cells tended to achieve higher proliferation rates than the control culture after a 48-h culture; however, dHF cells treated with colostrum 2 showed higher proliferation after 24 h (Figure 4b).

### 2.6. Scar Test Assay

**Figure 5:**
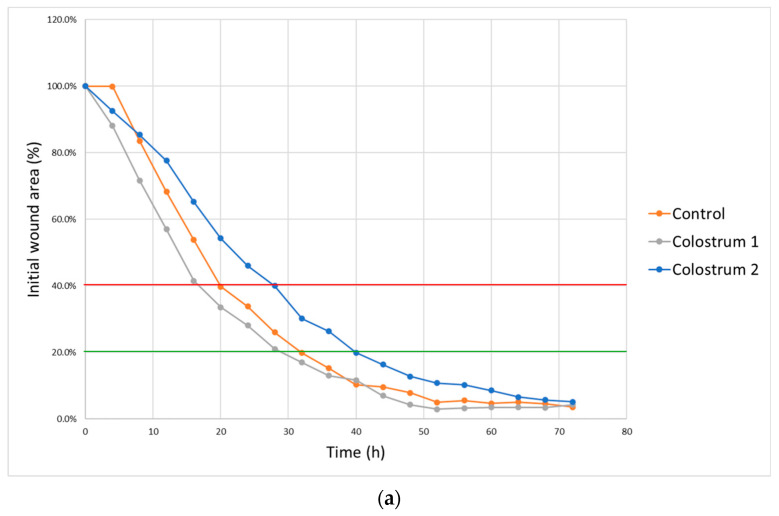

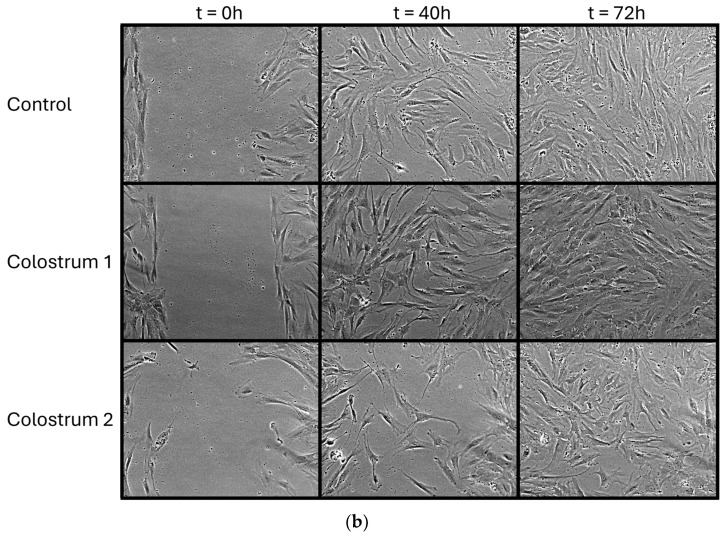
Scar test results for dHF cells stimulated with either colostrum 1 or colostrum 2 (n = 4). (**a**) dHF cells stimulated with colostrum 1 closed the scar to 40% of the initial scar area (red line) after 18 h of culture and 20% (green line) after 28 h of culture. Colostrum 2 did not increase the speed of dHF migration compared to control culture. (**b**) Representative photographs of wounds in three selected timepoints. In the scar test, colostrum 1 had a stronger impact on scar closure rate. It was shown that ovine colostrum has the potential to enhance wound healing processes in diabetic foot ulcers.

dHF cells cultured with colostrum 1 showed an increased wound closure rate. Cells treated with colostrum 1 proliferated and migrated faster than the cells in the control culture, covering 40% of the initial scar area after 16 h of culture and 20% of the initial scar area after 28 h. Meanwhile, cells treated with colostrum 2 covered 40% of the initial scar area in 28 h and 20% after 40 h of culture (Figure 5).

### 2.7. Gene Expression

**Figure 6:**
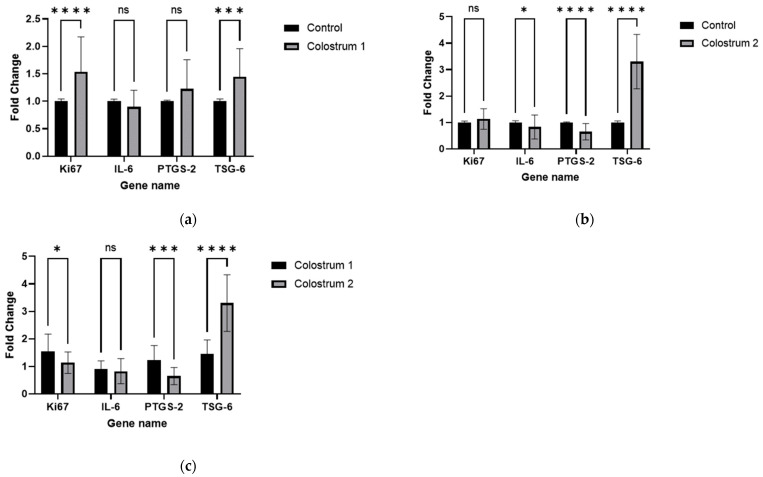
Expression of *Ki67*, *IL-6*, *PTGS-2*, and *TSG-6* in dHF after 48 h treatment with either colostrum 1 or colostrum 2 (n = 4). Panels: (**a**) dHF cells treated with colostrum 1 vs. control; (**b**) dHF cells treated with colostrum 2 vs. control; (**c**) colostrum 1 vs. colostrum 2. Statistical significance was determined in GraphPad program with the Mann–Whitney U-test with Holm–Šídák correction for multiple comparisons; **** *p* < 0.0001; *** *p* < 0.001; * *p* < 0.05; ns means no significance.

dHF cells cultured with colostrum 1 showed higher expression of *Ki67* (fold change = 1.53; *p* < 0.0001) and *TSG-6* (fold change = 1.44; *p* < 0.001) genes than the control culture (Figure 6a); in contrast, dHF cells cultured with colostrum 2 showed lower levels of *Il-6* (fold change = 0.82; *p* < 0.05), *PTGS-2* (fold change = 0.65; *p* < 0.0001) and significantly higher levels of *TSG-6* (fold change = 3.3; *p* < 0.0001) (Figure 6b).

The effects of colostrum 1 and colostrum 2 on the expression of genes related to proliferation (*Ki67*) and immune response (*IL-6*, *PTGS-2*, and *TSG-6*) were also compared in dHF cells from four patients. It was found that colostrum 1 significantly increased the expression of *Ki67* (*p* < 0.05) and *PTGS-2* (*p* < 0.001) genes compared to colostrum 2, while the latter demonstrated stronger stimulation of *TSG-6* expression (*p* < 0.0001). No significant differences in expression levels were observed for *IL-6* (Figure 6c).

Both colostrum 1 and 2 were found to enhance dHF proliferation rate in Presto Blue assay after a 48-h cell culture. However, differences in *Ki67* expression levels suggested that colostrum 1 has a higher potential to stimulate proliferation in dHF than colostrum 2 over longer periods. The observed differences in dHF response to colostrum 1 and 2 may stem from the different levels of LTF protein.

Colostrum 2 significantly increased the expression of *TSG-6* in dHF cell culture compared to controls and colostrum 1; this may suggest that colostrum 2 has anti-inflammatory properties [21]. The lack of any significant change and the lower levels of *IL-6* and *PTGS-2* expression may indicate that colostrum contains *TSG-6*-related stimulants.

## 3. Discussion

In this study, we analyzed the composition of sheep colostrum originating from Polish and Swiss animals. Further, the antioxidative properties of both colostrum were compared and their ability to inhibit the tyrosinase activity as well as the pro-proliferative impact on three types of human skin cells: neonatal epidermal keratinocytes, skin fibroblasts, and diabetic fibroblasts. Additionally, the pro-regenerative attributes of both colostrum were analyzed by scar tests and gene expression of diabetic fibroblasts.

### 3.1. Analysis of the Composition of Colostrum

The results of present study on protein and fat content in sheep colostrum differ from the results of other researchers. Wendorff et al. reported lower protein (11.8%) and fat (13.0%) content [22]. Different results were also observed in a study on ten sheep breeds, in which the protein and fat content ranged from 13.75% to 22.49% and from 4.04% to 13.64%, respectively [23]. In a study by Guiso et al., the average amounts of protein and fat in colostrum were 16% and 7.8%, respectively. In later milk, the protein and fat content dropped to 5.5% and 5.8% [11].

Such discrepancies could be attributed to various factors, including the form of colostrum (liquid or solid), the period after giving birth, sheep breeds, their diet, and breeding conditions. The content of ingredients in the liquid form of colostrum is lower than in its more concentrated form after lyophilization. Therefore, the solid form (freeze-dried) of our colostrum (1 and 2) may explain their much higher (by about 2–3 times) content of fat and protein than that reported in the literature [22,23]. Furthermore, our colostrums were obtained during the initial 12 h of lactation, a period when concentrations of substances like protein and fat are markedly higher than in later milk [22]. The protein and fat content can also vary significantly between breeds, and can be influenced by diet and environmental conditions [11,22,23].

Both colostrum samples demonstrated similar fatty acid profiles as noted in previous studies [11,24,25]. Guiso et al. report the following proportions of specific fatty acids: 37% monounsaturated fatty acids (MUFA), 58% saturated fatty acids (SFA), and 6% polyunsaturated fatty acids (PUFA) [11]. In comparison, the MUFA, SFA, and PUFA concentrations were 31.16%, 63.98%, and 4.86%, respectively, in colostrum 1 and 24.63%, 68.75%, and 6.62% in colostrum 2. A similar fatty acid profile was noted for bovine colostrum: 65.6% SFA and 27.5% MUFA [26].

Among the fatty acids, both in colostrum 1 and 2, oleic acid and palmitic acid were the dominant components with the highest contents: 22.11% and 23.46% and 37.41% and 37.14%, respectively. Topical application of oleic acid may compromise the integrity of the lipid barrier, as evidenced by research on olive oil, which is also predominantly composed of oleic acid [27,28,29]. Its topical application by healthy individuals significantly compromised the integrity of the stratum corneum and induced mild erythema [27].

Specific fatty acids such as CLA and long-chain PUFAs present in our samples are known for their anti-inflammatory and immunomodulatory properties [30,31,32]. The high levels of CLA in colostrum 2 may make it beneficial in reducing inflammation and helpful in the treatment of autoimmune diseases such as atopic dermatitis [33]. Long-chain polyunsaturated fatty acids (LC-PUFAs) have an inhibitory effect on the growth of *P. acnes* NCTC737 and *S. aureus* ATCC43300 bacteria [34]. This may indicate a beneficial effect of colostrum in the treatment of acne vulgaris.

### 3.2. Antioxidant Activity of Colostrum

Milk hydrophilic antioxidants include, among others, caseins, whey proteins (lactoferrin, β-lactoglobulin), peptides, minerals, vitamins, and nitrogen compounds of low molecular weight. Milk is also rich in antioxidant enzymes such as superoxide dismutase, lactoperoxidase, glutathione peroxidase, and catalase. Lipophilic antioxidants include conjugated linoleic acid (CLA), carotenoids, α-tocopherol, vitamins A and D3, phospholipids, and coenzyme Q10. The antioxidant capacity of milk varies between species of animal, diet, and the phase of lactation [35,36].

Among the tested milk samples of various mammals (sheep, cows, buffaloes, camels, and goats), sheep milk displayed the strongest DPPH radical scavenging capacity (%), amounting to 27.28%. This milk also had the highest content of total protein and fat [37].

Another study revealed the KGL3A-fermented sheep milk was a source of antioxidative peptides. The milk fractions showed antioxidative properties ranging from 18.4 to 34.6% (ABTS assay) [38].

In the present study, the 50% inhibition of ABTS and DPPH radicals by colostrum was observed for the following concentrations: 0.477 mg/mL and 0.988 mg/mL (colostrum 1) and 0.424 mg/mL and 0.806 mg/mL (colostrum 2), respectively.

### 3.3. Colostrum’s Impact on Tyrosinase Activity

Few studies have examined the potential of sheep colostrum and milk, or bovine colostrum, as tyrosinase inhibitory factors.

Nevertheless, one study compared tyrosinase inhibition by proteins, protein hydrolysates, individual peptides, and amino acids derived from industrial proteins (β-casein, α-lactalbumin, β-lactoglobulin, and ovalbumin). It was found that the peptides containing arginine and/or phenylalanine together with valine, alanine, and/or leucine were more effective inhibitors than those containing aspartic or glutamic acid residues [39]. Milk proteins such as k-casein and β-lactoglobulin are known to inhibit melanogenesis and their hydrolysates are antioxidant peptides [40]. Donkey milk is a promising skin whitening and antiphotoaging agent, showing a good inhibitory effect on melanin synthesis, tyrosinase activity, and related gene expression [41].

Although we may presume that either colostrum 1 or 2 are sources of whitening agents for prospective skin care, we did find in our research that colostrums have a slight tendency to decrease tyrosinase activity.

### 3.4. The Effect of Colostrum on Proliferation of Fibroblasts and Keratinocytes

Assessment of the effect of colostrum on stimulating the growth of the basic skin cells building the dermis and epidermis, fibroblasts and keratinocytes, as well as diabetic human fibroblasts (dHF) can serve as a model for effectiveness in supporting skin wound healing, its regenerative capacity, skin rejuvenation, and even delaying skin aging.

Bovine colostrum has been found to stimulate canine fibroblast growth in vitro. The effect of this colostrum was very similar to the effect of sheep colostrum 2 on human fibroblast cells in our study. The highest concentrations of bovine colostrum used, i.e., 0.3 mg/mL and 1 mg/mL, stimulated the growth of canine fibroblasts by 12% and 32% compared to the negative control after 48 h of incubation [42].

The protective effect of bovine colostrum on skin aging was determined based on telomere length and the rate of their shortening, this being a biomarker of aging, as well as by examining changes in the proliferation of human fibroblasts under standard conditions and under oxidative stress induced by H_2_O_2_. The results indicate that bovine colostrum can improve the condition of aging skin by increasing the rate of fibroblast cell proliferation and protecting against telomere length erosion, even in the presence of an oxidative stress factor [43].

Among three fractions (whey, casein, and fat globule) isolated from mare colostrum, the fat globule fraction stimulated fibroblasts strongest, probably due to the presence of lactadherin [44].

To date, the effects of any milk or colostrum on diabetic human fibroblasts (dHF) have not been studied. Our study found that proliferation on stimulated dHF cells ranged from 96.0% to 115.4%, with a lower rate compared to 1BR.3.N human skin fibroblasts, which reached up to 227% after 48 h. This may be related to the origin of dHF cells which were isolated from wounds of patients with complex metabolic disorders. These characteristics implicate the lower proliferative potential of dHF cells and can influence the outcome of the Presto Blue assay. It is possible that higher doses of colostrum would be required to achieve similar effects in dHF cells as in healthy cells, although this requires further research.

Moreover it should be noted that prolonged increases in fibroblast proliferation are not always desirable, as it can result in keloid scar formation [45]. Therefore, further and longer observations with colostrum are needed, especially in a clinical setting.

Different biological effects have been noted for keratinocytes. Colostrum 1 (24 h) was found to stimulate cell proliferation but only to a concentration of 1.5 mg/mL. In contrast, the remaining concentrations of colostrum 1 and all those of colostrum 2 inhibited keratinocyte proliferation at both incubation times. A similar inhibitory effect was demonstrated by bovine colostrum on human primary keratinocytes. The growth arrest was presumably caused by upregulation of cyclin-dependent kinase inhibitors that suggest the possibility of using colostrum for reducing the proliferation of keratinocytes in the treatment of skin diseases characterized by a disturbed skin barrier and altered differentiation [46].

### 3.5. Scar Test Assay

Colostrum 1 was found to exhibit potential to enhance wound healing processes in diabetic foot ulcers, probably due to its high levels of antioxidative agents and lactoferrin (LTF) protein, which may stimulate fibroblast proliferation and migration [47]. So far, no studies have examined the effect of colostrum from sheep or other animals on scar testing, which could open up an entirely new area for research. Taking into account the results of gene expression analysis, it could prove beneficial to test the dHF reaction to colostrum(s) following prior inflammatory priming.

Studies have compared the effectiveness of bovine colostrum powder dressings and conventional dressings for deep wounds. The results show that colostrum dressings were safe and promoted wound healing by shortening healing time and reducing pain as well as the number of dressing changes. Therefore, they can be used supportively in the treatment of deep wounds [48]. Bovine colostrum effectively supported wound healing, probably thanks to the presence of transforming growth factor (TGF-b), fibroblast growth factor (FGF), and insulin-like growth factor (IGF-1) [48,49].

### 3.6. Gene Expression

Very few studies have examined the effects of colostrum on gene expression and biological activity, and the existing ones have employed differing methodologies and sample types; as such, it is difficult to directly compare their findings with those of the present study. For example, most existing research has focused on bovine colostrum in liquid form, whereas the present study used freeze-dried sheep colostrum.

Research indicates that colostrum contains numerous bioactive components that can affect the gene expression and biological activity [50,51]. Two such components are the growth factors IGF-1 and EGF, which stimulate cell growth and proliferation. IGF-1 activates the IGF-1 receptor, inducing intracellular signaling that promotes DNA synthesis and cell division [52]. Immunomodulatory proteins such as lactoferrin (LTF) have anti-inflammatory, antibacterial, and immunomodulatory properties, inhibiting the production of pro-inflammatory cytokines such as IL-6 and increasing the expression of anti-inflammatory mediators [53,54]. Proline-rich polypeptides (PRPs) help regulate immune responses, reducing excessive inflammation [55]. Cytokines and chemokines, such as IL-10, can suppress the expression of pro-inflammatory cytokines such as IL-6 and TNF-α [51,56]. Transforming Growth Factor Beta (TGF-β) regulates cell proliferation and differentiation; it also plays a key role in wound healing by stimulating fibroblast proliferation and extracellular matrix protein synthesis [50,57].

Colostrum 1 strongly increased *Ki67* expression, indicating a strong proliferative effect. Although colostrum 2 also increased cell proliferation, its effect on *Ki67* expression was less pronounced than that of colostrum 1; this may suggest differences in the bioactive composition of the two samples. Those differences in expression are coherent with the scar test assay results, where dHF cells stimulated with colostrum 1 were first to reach the 40% and 20% breakpoints. Even though no drastic differences between colostrum samples were observed over the course of the full-length observation, this initial burst in migration and proliferation speed of dHF cells, together with increased *Ki67* expression, suggests that colostrum 1 has greater proliferation-promoting effects.

The observed reduction in *IL-6* expression by colostrum 2 indicates potential anti-inflammatory effects, which is supported by the significant decrease in *PTGS-2* expression. *PTGS-2* (*COX-2*) is an enzyme associated with the inflammatory process, and its reduction suggests a coordinated anti-inflammatory response [58,59].

The increase in *TSG-6* expression by colostrum 1 also suggests beneficial anti-inflammatory effects. *TSG-6* is a protein that protects tissues from inflammatory damage. Nevertheless, colostrum 2 yielded an exceptionally high increase in *TSG-6* expression, further underscoring the higher anti-inflammatory properties of this colostrum, and possibly the presence of stronger *TSG-6* stimulants.

Previous research has demonstrated that bovine colostrum contains growth factors such as IGF-1 and EGF, which can stimulate the proliferation of intestinal epithelial cells, supporting observations of increased *Ki67* expression by colostrum 1 [50]. Additionally, bovine colostrum has been found to reduce the production of pro-inflammatory cytokines such as IL-6, which is consistent with the results regarding colostrum 2 and its anti-inflammatory effects [51].

Since dHF can be burdened with diabetes-related “metabolic memory”, the increased expression of anti-inflammatory genes, such as *TSG-6*, and the decreased expression of the pro-inflammatory *IL-6* gene may suggest that colostrum has a beneficial effect on the dysregulated inflammatory properties of dHFs [60]. In addition, a fibroblast population subcluster in diabetic ulcer wound tissue demonstrated increased *TSG-6* expression which was strongly related with positive healing processes [61].

In summary, colostrum 1 shows stronger proliferative effects (higher stimulation of *Ki67*), while colostrum 2 exhibits stronger anti-inflammatory properties (higher stimulation of *TSG-6*). These differences may result from variations in protein composition, including levels of lactoferrin and other bioactive components, and may indicate different future applications for either colostrum. Those pilot observations regarding the reaction of dHF to stimulation with colostrum(s) can be fully understood and interpreted in combination with the proteomic analysis of colostrum. Such analyses are currently being conducted by the authors and will be published in the near future. However, the properties shown in this study indicate that colostrum 1 could have higher potential in acute wound healing applications, whereas colostrum 2 could find use in the management of chronic wounds, thanks to their ability to activate the expression of different genes in dHF cells.

## 4. Materials and Methods

### 4.1. Colostrum Samples

Sheep colostrum from two sources, Polish and Swiss, was used in the research.

Colostrum 1 (Polish origin) was collected from Lacaune sheep living on a farm in central Poland. Colostrum 1 was collected during the first 12 h of sheep lactation and immediately frozen. The tested batch was a mixture of colostrum from approximately 20 sheep. It was stored at—22 °C for a few days and then freeze-dried. Colostrum 1 was not pasteurized.

Colostrum 2 (Swiss origin) was obtained as a commercially available product: Swiss Sheep Colostrum Vivienne Swiss Formula. Colostrum 2 is sheep colostrum from Swiss-controlled organic farms; it is collected only within the first 12 h after birth, pasteurized by the manufacturer at temperatures up to 63 °C, and freeze-dried.

### 4.2. Freeze-Drying

Frozen colostrum 1 was subjected to sublimation drying (lyophilization) using a FreeZone 18L apparatus (Labconco, Kansas City, MO, USA). The process began at a temperature of −40 °C for the first hour, then the shelf temperature was gradually increased to 40 °C at a rate of 0.05 °C/min and maintained under these conditions for the next 48 h. The process pressure was kept constant at 0.230 mBar.

### 4.3. Fat Analysis

The fat content of sheep colostrum was determined using the Soxhlet method. Fat was extracted using a Büchi B-811 extraction system (Büchi Labortechnic AG, Flawil, Switzerland). A 3 g sample of sheep colostrum was extracted for 40 extraction cycles using hexane as a solvent [62].

### 4.4. Protein Analysis

Approximately 1 g of sheep colostrum was weighed into mineralization flasks, two Kjeldahl copper catalyst tablets (3.5 g K₂SO₄ + 0.4 g CuSO₄∙5H₂O) were added, and 25 mL of concentrated H₂SO₄ was added, followed by sample mineralization. Protein nitrogen was distilled and titrated with a standardized HCl solution using the Kjeltec 9 system (FOSS; Hilleroed, Denmark). The protein conversion factor used was N*6.25 [63].

### 4.5. Fatty Acid Analysis

The extracted lipids (approximately 50 mg) were placed in a hydrolysis tube; 4 mL of 0.5 M NaOH solution in methanol and 4 mL of BF3 (14% solution in methanol) were added. The solution was heated in a heating block for 0.5 h at 70 °C. After the elapsed time, the obtained esters were extracted with 6 mL of hexane. The hexane layer was dried through a layer of anhydrous magnesium sulfate, evaporated to dryness in a stream of nitrogen, and the residue was dissolved in 1.5 mL of hexane and subjected to chromatographic analysis (Agilent 6890N Series GC), using a 88% cyanopropyl and 12% aryl polysiloxane column (HP-88, 100 m 0.25 mm 0.25 μm) and MS detector (Agilent 5973 MS Detector).

### 4.6. ABTS Free Radical Scavenging Activity Assay

The antioxidant activity assay was carried out using the slightly modified ABTS [2,2′-azino-bis(3-ethylbenzothiazoline-6-sulphonic acid) diammonium salt] radical cation decolorization method [64]. The ABTS^●+^ stock solution was prepared by mixing equal volumes of 7.00 mM ABTS solution [96.02 mg (0.1750 mmol) in 25 mL water/methanol (1:1)] with 2.45 mM potassium persulfate solution: 16.56 mg (0.06126 mmol) in 25 mL water/methanol (1:1). This mixture was allowed to stand in the dark at room temperature for 16 h before use. The ABTS^●+^ stock solution (0.25 mL) was then diluted with water (5 mL) to give an absorbance of the negative control about 0.7 at 734 nm.

The measurement was performed for the following colostrum concentrations: 0.25 mg/mL, 0.50 mg/mL, 0.75 mg/mL, and 1.00 mg/mL. The highest colostrum concentration of 1 mg/mL was obtained by dissolving 100 mg of colostrum in 100 mL of distilled water. The suspension was heated at 30 °C for about 30 min with constant stirring. Lower concentrations were obtained by appropriate dilution of a solution with a concentration of 1 mg/mL with distilled water.

Following this, 5 mL of the test solution at a particular concentration was combined with 0.25 mL of the ABTS^●+^ stock solution and then the absorbance of the mixture was measured at 734 nm (Shimadzu Spectrophotometer UV-1800, Kyoto, Japan). The absorbance of each solution was assayed after 10 min, 20 min, 30 min, and 40 min. Ascorbic acid was used as a standard (assayed only after 10 min). The results were expressed as EC_50_, the concentration of sample at which 50% of maximum scavenging activity was recorded, which was calculated from the calibration curve.

### 4.7. DPPH Radical Scavenging Activity

The DPPH [2,2-diphenyl-1-picrylhydrazyl] (Merck Life Science Sp.z.o.o.; Poznan; Poland) radical scavenging activity was determined as reported previously [65,66,67]. For this purpose, 10 mL of the colostrum sample at various concentrations (0.25; 0.5; 0.75; and 1.0 mg/mL) was mixed with 10 mL of an ethanol solution of DPPH (125 mM) in a 1:1 ratio. The mixtures were incubated for 60 min in darkness at room temperature. For the control, distilled water was used rather than the sample. The absorbance of solution was read at 517 nm using a Shimadzu spectrophotometer UV-1800 (Kyoto, Japan). The DPPH radical scavenging activity (%) was calculated according to this formula:

$$DPPH\;inbibition\;(\%)=\frac{A-(B-C)}{A}\times 100\%$$

where A indicates absorbance of DPPH solution without the colostrum sample; B indicates absorbance of DPPH solution with the colostrum sample; and C indicates absorbance of the colostrum sample.

### 4.8. Inhibition of Tyrosinase Activity

Tyrosinase inhibition was measured according to two reported methods [68,69]. A starting tyrosinase solution was prepared by adding 0.5 mL of fungal tyrosinase (167 U/mL; Merck Life Science Sp.z.o.o., Poznan, Poland) the solution containing 25.45 mL of 0.1 M phosphate buffer (pH 7.0). Following this, 1.5 mL of tyrosinase solution, 1 mL of colostrum solution (1 mg/mL), and 3.5 mL of phosphate buffer were mixed to form the appropriate solution. The colostrum concentration of 1 mg/mL was obtained by dissolving 100 mg of colostrum in 100 mL of distilled water. The suspension was heated at 30 °C for about 30 min with constant stirring. The mixture was then incubated for 5 min at 30 °C. After this time, 5 mL of 3,4-dihydroxyphenyl-L-alanine (L-DOPA) was added to initiate the enzymatic reaction. Absorbance at 477 nm was measured for 20 min to monitor L-DOPA formation by Shimadzu spectrophotometer UV-1800 (Kyoto, Japan). Ascorbic acid (1 mg/mL) served as a positive control that was used for comparison. The inhibition rate was calculated as follows:

$$Tyrosinase\;inbibition\;(\%)=\frac{\left[(A-B)-(C-D\right)}{A-B}\times 100\%$$

where A indicates the absorbance of the solution with tyrosinase without sample, B indicates the absorbance of the solution without sample and tyrosinase, C indicates the absorbance of the solution with sample and tyrosinase, and D indicates the absorbance of the solution with sample and without tyrosinase.

### 4.9. Proliferation of Fibroblasts and Keratinocytes

The influence of colostrum on cell viability was tested using 1BR.3.N human skin fibroblast (cat. no. 90020508, ECACC) and human neonatal epidermal keratinocytes (cat. no. SCCE020, Merck Life Science Sp.z.o.o., Poznan, Poland). The cells were cultured at 37 °C and 5% CO_2_ in a humidified atmosphere, with the usage of appropriate media recommended by manufacturer.

For the viability assessment, the cells were seeded on 96-well plates, 8000 cells per well; six replicates were made for each concentration in full culture medium. The next day, the medium was changed to starving with an appropriate colostrum concentration. Colostrum was weighed and pre-dissolved in MEM medium (Gibco, Thermo Fisher Scientific, Waltham, MA, USA) (without FBS) for 2 mg/mL working solution; this was vortexed and incubated for approx. 30 min in a water bath at 37 °C to form a homogeneous suspension. This was then filtered through syringe filters with a pore diameter of 0.22 µm. After filtration, dilutions of both tested batches of colostrum in culture medium were prepared—0.1; 0.25; 0.5; 0.75; 1; 1.25; 1.5; 1.75; and 2 mg/mL—and applied to cells.

The MTT (3-[4,5-dimethylthiazol-2-yl]-2,5 diphenyl tetrazolium bromide) (Thermo Fisher Scientific, Naarden, The Netherlands) viability assay was performed after 24 and 48-h incubation. Briefly, the cell culture medium was extracted and replaced with 110 µL of MTT solution in PBS (Thermo Fisher Scientific, Naarden, The Netherlands). After incubation for three hours at 37 °C, the MTT solution was extracted and 100 µL DMSO (Applied Biological Materials Inc., Richmond, BC, Canada) was added. The formazone crystals were allowed to dissolve for 10 min at room temperature on the cradle, and the absorbance was read at 570 nm with an Infinite F50 Tecan plate reader (Life Sciences, Mannedorf, Switzerland).

The raw absorbance measures were normalized against negative control without cells and the cellular viability was presented as a percentage of positive controls, i.e., wells with cells but without colostrum. The significance of the difference in the viability of the treated and untreated cells was analyzed with the two-sided *t*-test. The difference was considered statistically significant at *p* < 0.05.

### 4.10. Diabetic Human Fibroblast Culturing

Diabetic human fibroblasts (dHF) were isolated from tissue samples collected from wounds of four patients with diabetic foot ulcers. All patients providing samples provided their informed consent to provide material for the research study (approval no. KB/128/2019 and KB/3/A2021).

The samples were expanded in standard dHF medium consisting of high-glucose DMEM medium (Gibco, Thermo Fisher Scientific, Waltham, MA, USA) supplemented with 10% fetal bovine serum (Gibco, Thermo Fisher Scientific, Waltham, MA, USA) and 1% antibiotic–antimycotic solution (Corning, Amsterdam, -The Netherlands) in standard culture condition (5%CO_2_/37 °C). Then, cells were harvested after appropriate culturing time depending on the planned analyses.

### 4.11. Diabetic Human Fibroblast Proliferation Assay

For the proliferation assay, the cells were seeded on the 96-well plate (20,000 cells/cm^2^) and cultured for three days. Then, the culture medium was changed to dHF medium supplemented with 1 mg/mL of either colostrum 1 or colostrum 2. Standard dHF medium was used as a control. The cells were cultured for 48 h, during which time a proliferation assay was performed twice, i.e., after 24 h and 48 h. For the proliferation assay, Presto Blue Cell Viability reagent (Invitrogen, Thermo Fisher Scientific, Waltham, MA, USA) was used as a 10% addition to dHF standard medium. The cells were incubated for one hour at 37 °C. After the incubation, the medium was transferred to a new 96-well plate and the fluorescence was measured (excitation/emission = 540 nm/590 nm) on a plate reader (Spark, Tecan, Switzerland). To calculate the proliferation rate, the mean blank value was subtracted from the fluorescence intensity values. The mean value of fluorescence intensity was calculated for each cell sample separately. To calculate the proliferation rate, the fluorescence values for stimulated cells were divided by the mean of the fluorescence of the controls and are shown as the percentage of the proliferation rate.

### 4.12. Scar Test

For the scar test, the dHF cells were plated onto 24-well plates using Culture-Inserts 2 Well (ibidi GmbH, Gräfelfing, Germany)—32,000 cells/inserts well. The cells were cultured for three days to allow them to adhere to the culture plate. On day three, the Culture-Inserts were removed and the culture medium was changed to either standard dHF medium, medium supplemented with 1 mg/mL of colostrum 1, or colostrum 2. The cells were then placed in a Nikon Eclipse Ti (Nikon Instruments Inc., USA) inverted microscope with the live chamber providing the needed culture conditions—5% CO_2_, 37 °C, and humidity. Scar images were acquired at four-hour intervals for a total observation period of 72 h. Each scar had up to four acquisition points set. Only a brightfield channel was used.

#### Image Analysis

Scar images were analyzed using ImageJ software [70] with the Wound Healing Size Tool macro [71]. Images were analyzed only on the brightfield channel. Before using the Wound Healing Size Tool, the brightness was adjusted to reduce the noise and improve reproducibility between the stack images. This adjustment was performed using auto adjustment set on the darkest frame, and the following macro was used:

run(“Bandpass Filter...”, “filter_large = 40 filter_small = 3 suppress = None tolerance = 5 autoscale saturate process”); run(“Subtract Background...”, “rolling = 50 light stack”);

run(“Make Binary”, “method = Default background = Default calculate black”).

The acquired results were collected and analyzed collectively for each well. The wound closure rate was calculated as the percentage of the no-cell area at time T = 0 h.

### 4.13. Gene Expression Analysis

The dHF cells from four patients were plated in T25 flasks and cultured in standard dHF medium. On day three after plating, the culture medium was changed to either fresh standard dHF medium or dHF medium supplemented with 1 mg/mL of colostrum 1 or colostrum 2. The cells were incubated at 37 °C, 5%CO_2_ for 48 h. Then, cells were collected using Accutase Cell Detachment Solution (Becton Dickinson, MA, USA). The cell count and viability were assessed on the ADAM automated cell counter (NanoEnTek Inc., Seoul, Republic of Korea). The cells were then washed in PBS (Gibco, Thermo Fisher Scientific, Waltham, MA, USA) and centrifuged at 12,000× *g* for one minute. The PBS was discarded and the dry cell pellets were quickly placed at −80 °C.

#### 4.13.1. RNA Isolation and cDNA Synthesis

The total RNA was isolated from frozen cell pellets using a High Pure RNA Isolation Kit (Roche, Germany) according to the manufacturer’s protocol. RNA concentration was assessed with a NanoDrop spectrophotometer (Thermo Fisher Scientific, Waltham, MA, USA). cDNA templates were synthesized using Maxima First Strand cDNA Synthesis Kit for RT-qPCR (Thermo Fisher Scientific, Waltham, MA, USA) according to the manufacturer’s protocol.

#### 4.13.2. qPCR

The qPCR reaction was performed using 30 ng of cDNA template per well. Each sample was analyzed in triplicate using TaqMan™ Fast Advanced Master Mix for qPCR (Applied Biosystems, Thermo Fisher Scientific, Waltham, MA, USA) with the following Taq Man assays: as housekeeping gene, *18S rRNA* (Hs99999901_s1); as genes of interest, *Ki67* (Hs01032443_m1), *IL-6* (Hs00174131_m1), *PTGS-2* (Hs00153133_m1), and *TSG-6* (Hs00200180_m1) (Applied Biosystems, Thermo Fisher Scientific, Waltham, MA, USA). The single plex reaction was carried out on 7500 Real-Time PCR thermalcycler (Applied Biosystems, Thermo Fisher Scientific, Waltham, MA, USA) using a fast cycle program.

#### 4.13.3. qPCR Data Analysis

The data were collected from Fast 7500 software 7500 Fast as Ct values and then exported. Ct values were used to calculate fold changes for each gene/sample pair according to the 2^−ΔΔCt^ method [72,73].

### 4.14. Statistical Analysis

For dHF proliferation assay, the standard error of measurement (SEM) was calculated for each patient. The significance of the differences between test and control samples were determined based on Fisher’s test and Student’s *t*-test, with the *p*-value threshold = 0.05. The same procedures were carried out for all four cell samples as one group.

qPCR results were analyzed with GraphPad Prism 9 (GraphPad Software, USA). Tests used Mann–Whitney U with a set *p*-value threshold *p* = 0.05; data were presented as mean ± standard deviation (SD).

## 5. Conclusions

Due to its biological properties, sheep colostrum has the potential to be used as an active ingredient in cosmetic and medicinal preparations for skin care and treatment. Sheep colostrum may exhibit anti-aging, regenerative, and anti-inflammatory effects on the skin and accelerate wound healing.

The possible rejuvenating and regenerative effects of colostrum on the skin, as well as the delaying of aging processes, may be predicted by its antioxidant properties, high ability to stimulate the growth of fibroblasts, and moderate of keratinocytes. Cells divisions slow down in (photo)aging skin, so providing colostrum ingredients that could stimulate dermal proliferation is crucial for skin regeneration.

Based on in vitro tests performed on dHF, it can be concluded that sheep colostrum may also have a beneficial effect on wound healing processes in diseased skin. It was shown in this study that depending on its origin, colostrum stimulated in vitro diabetic human fibroblast (dHF) proliferation (*Ki67*) and migration, as well as increased the expression of anti-inflammatory genes (*TSG-6*) while decreasing the expression of the pro-inflammatory *IL-6* gene. However, further studies, e.g., proteomic analysis or animal model observations, should be performed to confirm those results.

## Figures and Tables

**Table 1:** Content (%) of fat and protein in lyophilised colostrum 1 and 2.

	Colostrum 1			Colostrum 2		
	M	SD	SE	M	SD	SE
Fat (%)	38.05 ^a^	0.50	0.17	26.63 ^a^	0.75	0.25
Protein (%)	34.14 ^b^	0.23	0.12	43.68 ^b^	0.37	0.18

M—mean value; SD—standard deviation, SE—standard error; ^a^ n = 9; ^b^ n = 4 (n—number of independent measurements).

**Table 2:** Fatty acid content (%) in lyophilized colostrum 1 and 2.

			Colostrum 1			Colostrum 2		
			M	SD	SE	M	SD	SE
Caprylic acid	CH_3_(CH_2_)_6_COOH	C8:0	0.00	0.00	0.00	0.36	0.02	0.01
Capric acid	CH_3_(CH_2_)_8_COOH	C10:0	0.00	0.00	0.00	1.35	0.06	0.03
Lauric acid	CH_3_(CH_2_)_10_COOH	C12:0	2.34	0.39	0.23	2.59	0.19	0.11
Myristic acid	CH_3_(CH_2_)_12_COOH	C14:0	11.17	1.17	0.68	13.04	0.65	0.38
Pentadecylic acid	CH_3_(CH_2_)_13_COOH	C15:0	1.09	0.07	0.04	1.52	0.45	0.26
Palmitic acid	CH_3_(CH_2_)_14_COOH	C16:0	37.41	0.16	0.09	37.14	0.96	0.56
Palmitoleic acid	9-cis-Hexadecenoic acid	C16:1	2.04	0.30	0.17	1.17	0.02	0.01
Margaric acid	CH_3_(CH_2_)_15_COOH	C17:0	1.32	0.28	0.16	0.89	0.16	0.09
Stearic acid	CH_3_(CH_2_)_16_COOH	C18:0	10.65	0.80	0.469	11.86	0.72	0.41
Oleic acid	cis-9-octadecenoic acid	18:1 (n-9)	29.11	1.26	0.73	23.46	0.58	0.33
Linoleic acid (LA)	all-cis-9,12-octadecadienoic acid	18:2 (n-6)	2.98	0.18	0.11	3.97	0.22	0.13
Conjugated linoleic acid (CLA)	(9Z,11E)-Octadeca-9,11-dienoic acid	CLA	0.76	0.06	0.04	1.64	0.05	0.03
Alpha-linolenic acid (ALA)	all-cis-9,12,15-octadecatrienoic acid	18:3 (n-3)	1.12	0.04	0.02	1.01	0.042	0.022
		SFA	63.98	1.11	0.64	68.75	0.78	0.45
		MUFA	31.16	0.98	0.56	24.63	0.59	0.34
		PUFA	4.86	0.15	0.08	6.62	0.21	0.12

M-mean value; SD-standard deviation, SE-standard error; (n = 3).

**Table 3:** Antioxidant activity of colostrum 1 and colostrum 2 expressed as EC_50_ [mg/mL]. The values represent the mean ± standard deviation of three independent measurements (n = 3).

	ABTS				DPPH
	EC_50_ [mg/mL] ± SD				EC_50_ [mg/mL] ± SD
	10 min	20 min	30 min	40 min	30 min
Colostrum 1	0.727 ± 0.051	0.574 ± 0.034	0.515 ± 0.048	0.477 ± 0.048	0.988 ± 0.137
Colostrum 2	0.625 ± 0.051	0.512 ± 0.033	0.455 ± 0.043	0.424 ± 0.053	0.806 ± 0.155
Ascorbic acid	0.0053 ± 0.0007				0.0026 ± 0.0003

**Table 4:** Inhibition of tyrosinase activity (%) by colostrum 1 and 2.

Colostrum 1	Colostrum 2	Ascorbic Acid
M ± SD	M ± SD	M ± SD
23.4% ± 0.6%	7.0% ± 2.0%	96.0% ± 2.2%

M—mean value; SD—standard deviation; (n = 4).

## Data Availability

The original contributions presented in the study are included in the article; further inquiries can be directed to the corresponding authors.
